# Determination of Compressibility and Relaxation Behavior of Yeast Cell Sediments by Analytical Centrifugation and Comparison with Deposit Formation on Membrane Surfaces

**DOI:** 10.3390/membranes12060603

**Published:** 2022-06-10

**Authors:** Maria E. Weinberger, Ulrich Kulozik

**Affiliations:** Chair of Food and Bioprocess Engineering, TUM School of Life Sciences, Technical University of Munich, Weihenstephaner Berg 1, 85354 Freising, Germany; ulrich.kulozik@tum.de

**Keywords:** bio-suspensions, BSA, deposit layer formation, downstream processing, microfiltration, saccharomyces

## Abstract

Separation of cells from produced biomolecules is a challenging task in many biotechnological downstream operations due to deposit formation of the retained cells, affecting permeation of the target product. Compression and relaxation behavior of cell deposits formed during filtration are important factors affecting operational performance. The determination of these factors by flux or pressure stepping experiments is time- and labor-intensive. In this work, we propose a screening method by analytical centrifugation, which is capable of detecting small differences in compression and relaxation behavior induced by milieu changes, using a model system comprised of washed and unwashed yeast cells in the presence or absence of bovine serum albumin as a model target protein. The main effects observed were firstly the impact of pH value, affecting interaction of bovine serum albumin and yeast cells especially close to the isoelectric point, and secondly the effect of washing the yeast cells prior to analysis, where the presence of extracellular polymeric substances led to higher compressibility of the deposited cells. By comparing and validating the obtained results with dead-end filtration trials, the stabilizing role of bovine serum albumin in deposits formed at low pH values due to interactions with the yeast cells was underlined.

## 1. Introduction

The formation of deposits is the major drawback of pressure-driven membrane-based separation processes. The characterization of the formed deposit layer is crucial for understanding the impact of process conditions and fouling mitigation strategies on micro- and ultrafiltration performance [1]. One property of interest is the compressibility of deposited material. The compressibility influences the porosity of the deposit layer and thus determines the increasing specific filtration resistance at increasing transmembrane pressures and the retention or transmission of solutes [2,3]. The compressibility of foulants therefore determines the optimum transmembrane pressure range for effective filtration operation [4]. In addition to that, the reversibility of compression, i.e., the relaxation upon pressure release, has an influence on the effectiveness of fouling mitigation, for instance by intermittent relaxation in dead-end filtration mode [5] or by applying pressure pulses during crossflow filtration [6,7].

So far, compressibility of deposits build up during micro- and ultrafiltration is mostly determined by single filtration experiments at different pressures [8,9] or by pressure or flux stepping experiments [3,10]. However, this is a time and work-intensive approach. Loginov, et al. [11] were first to develop a high throughput method based on analytical centrifugation, which is capable of assessing compressibility of different particulate matter simultaneously with only small sample volume and is thus an alternative for flux stepping experiments at screening stage. This method was applied by these authors for comparing compressibility of inorganic and chemically inert material such as calcium carbonate and kaolin as well as yeast suspensions without considering interaction of different materials in the sediments. A link to deposit formation during membrane filtration for systems comprised of biological material such as cell-protein mixtures was not reported. Also, the rather small differences for suspensions with the same particles at different physicochemical conditions, such as pH value, which nonetheless can have drastic impact on filtration performance have so far been only assessed by flux stepping experiments.

The reversibility of deposit compression during microfiltration is usually determined by a series of increasing pressure steps followed by steps with decreasing pressure [12,13,14]. This approach is a complex procedure with one sample at a time, and an alternative screening method would be beneficial. We therefore developed a method able to assess compressibility as well as reversibility of compression of biological particulate matter mixed with biogenic macromolecules, based on the previously described multistep centrifugal consolidation method proposed by Loginov, et al. [15]. As a model particle, yeast cells were used, an established model foulant also used in other microfiltration studies [16,17,18,19], which are also of economic relevance in biotechnological applications, such as production of biopharmaceuticals and biorefinery. Yeast cells were washed and suspended in buffers with different pH and were additionally prepared as an unwashed suspension in order to vary the milieu and the interaction of components to obtain deposits with different compressibility and relaxation behavior.

The following gives a short overview over the current knowledge on specific resistance, compressibility and reversibility of deposits formed from yeast cells during microfiltration and the most important influential factors.

Ní Mhurchú and Foley [20] systematically investigated the impact of different pH, salt concentration, yeast concentration, transmembrane pressure and membrane resistance during dead-end microfiltration of a yeast suspension. The correlation of specific resistance by an artificial neural network showed that pH value and transmembrane pressure had the highest relative effect on specific filtration resistance. The specific filtration resistance increased in the following order of pH value: pH 4, pH 3, pH 5, pH 6. Furthermore it was found that the presence of extracellular polymeric substances, i.e., the use of unwashed yeast cells, not only increased the overall resistance, but also reduced the reversibility of fouling [21,22].

Ye and Chen [12] investigated the fouling behavior as well as the reversibility of deposit layers formed from washed yeast cells and yeast cells in presence of BSA in flux stepping microfiltration experiments. The washed yeast cells formed a reversible cake with only low filtration resistance. The addition of BSA, however, increased the irreversibility. The lowest compressibility was found at pH 3, where the yeast cells are only slightly negatively charged, whereas BSA is positively charged, which led to a stable and almost incompressible cake. However, at pH 3, also the observed reversibility was low. Foley [23] attributed the lower specific resistance at lower surface charge (pH closer to the isoelectric point of yeast cells) to the occurrence of aggregation, which leads to an open-porous deposit structure due to the supporting effect of the aggregates.

In summary, some basic aspects of deposited structures on membrane surfaces from yeast cell-BSA mixtures are known. Other studies initiated the development of a centrifugal method for the characterization of sediments, which appears as an attractive method for the evaluation of the filtration behavior of particulate suspensions. The combination of both methods by means of a head-to-head comparison is lacking so far for complex systems considering interactive effects between different biogenic components, such as cells and proteins. We chose to use yeast cells and BSA, as both are frequently used model components in filtration studies and well characterized on a cellular/molecular properties level as well as regarding their fouling tendencies. The aim of this study therefore was to study the compressibility and relaxation behavior of centrifugal sediments of yeast cells in a head-to-head comparison with deposits formed on membrane surfaces. The use of suspensions of washed yeast cells at different pH as well as an unwashed yeast suspension should result in deposits with different compressibility and relaxation behavior. We hypothesize that these differences can be detected by analytical centrifugation and that physicochemical interaction between cells and protein also affects the microfiltration behavior of the model suspension in terms of filtration resistance as well as transmission of the dissolved protein.

## 2. Materials and Methods

### 2.1. Preparation of the Cell Suspension

The yeast cell suspension was prepared using fresh baker’s yeast (F.X. Wieninger GmbH, Passau, Germany). As a washing step, the yeast was suspended in buffer in a volume ratio of 1:1 and subsequently centrifuged at 6000× *g* for 10 min in order to ensure a sufficient medium replacement. The supernatant was discarded and the pellet was resuspended in buffer at the desired dry matter content of 5 and 10%. The buffer was a 0.1 M acetate buffer with different pH value in the range from pH 3 to pH 6, prepared from acetic acid and sodium acetate trihydrate (AppliChem GmbH, Darmstadt, Germany). Unwashed yeast suspension was prepared by dissolving fresh yeast in deionized water, which led to a pH value of 4.5. The dry matter content was set to 5 and 10% for washed and unwashed suspensions. The focus of this work is on the results of 10% dry matter content suspensions, due to the higher resolution during analytical centrifugation based on higher sediment beds. The results for both dry matter contents were alike. For some experiments, BSA (Sigma Aldrich, St Louis, MO, USA) was added to the suspension at a concentration of 0.8 g·l^−1^. The chosen concentration is low enough to enable complete dissolving and high enough to enable accurate quantification. The experiments chosen represent suspensions with pH values above, below and close to the isoelectric point of BSA. In order to quantify the shielding of surface charges in different buffers, the debye length $\lambda_{D}$ of the buffers was calculated according to Equation (1), where $\varepsilon_{0}$ is the vacuum permittivity, ε is the dielectric constant of the suspending fluid (ε=80 for water), $k_{B}$ is the Boltzmann constant, T is the temperature, $N_{A}$ is the Avogadro number, e is the elementary charge and I is the ionic strength.

(1) $$\lambda_{D}=\sqrt{\frac{\varepsilon_{0}\cdot \varepsilon \cdot k_{B}\cdot T}{2\cdot N_{A}\cdot e^{2}\cdot I}}$$

### 2.2. Analytical Methods

The dry matter content of the suspensions was measured by a SMART 6 drying balance (CEM Corporation, Matthews, NC, USA), drying the sample by microwave-assisted heating. The density was determined using a temperature-controlled oscillating U-tube density meter DMA 4100 M (Anton Paar, Graz, Austria). The cell size distribution was measured with a mastersizer 2000 equipped with a hydro 2000S dispersion unit (Malvern Pananalytical Ltd., Malvern, UK) and did not vary between the different buffers. Thus, cell lysis did not significantly take place, even at low pH values.

For solid particles, the particle volume fraction Φ can be calculated considering the solid weight fraction of the suspension $c_{s}$, the particle density $\rho_{p}$ and liquid density $\rho_{l}$ [15]. Living cells, however, contain intracellular water, which has to be considered. Furthermore, salts solved in the liquid phase also add up to the solid weight fraction. We therefore calculated Φ according to Equation (2), considering solid weight fraction c and density ρ of the overall suspension (index s), the yeast particles (index p) and the surrounding liquid (index l). The particle density $\rho_{p}$ was set to 1130 kg·m^−3^ for yeast cells, according to Loginov, Lebovka and Vorobiev [11].

(2) $$\Phi =\frac{c_{s}\cdot \rho_{s}-c_{l}\cdot \rho_{l}}{c_{p}\cdot \rho_{p}-c_{l}\cdot \rho_{l}}$$

### 2.3. Compressibility Analysis

**Figure 1:**
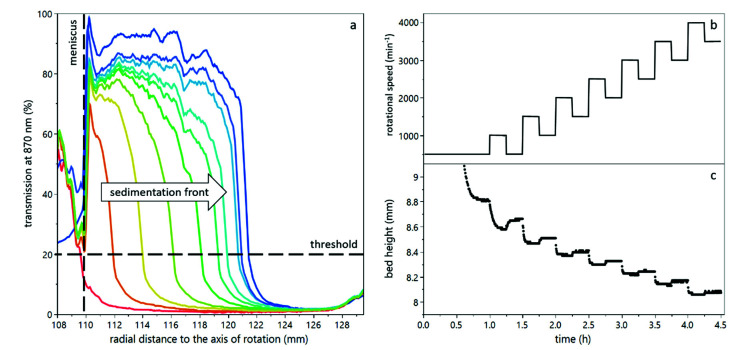
Working principle and set centrifugation steps during analytical centrifugation. The transmission of light as a function of radial position can be used to determine the sediment bed height (**a**). The rotational speed was increased stepwise with relaxation steps between (**b**) in order to determine compressibility as well as relaxation of the sediment in terms of the sediment bed height (**c**).

The initial sedimentation and subsequent compression and consolidation of the deposited yeast cells was analyzed with an analytical centrifuge, called LUMiFuge (LUM GmbH, Berlin, Germany). This analytical centrifuge measures the transmission of light at 870 nm wavelength through the sample cuvette during centrifugation as a function of the radial position. Figure 1a shows an exemplary transmission profile as a function of centrifugation time. The progression of the sedimentation front to the cuvette bottom can be observed. The resulting bed height can provide information about the degree of compression of the sediment. By increasing the rotational speed, the compressive forces were increased. In order to understand the compression behavior and the reversibility of compression, i.e., the relaxation, a stepwise increase of rotational speed with relaxation steps in between was conducted (see Figure 1b). Each step was kept long enough to reach an equilibrium (see Figure 1c). Thus, an initial sedimentation step at 500 min^−1^ was kept for 60 min, followed by upwards steps of 1000 min^−1^ and downwards steps of 500 min^−1^, each kept for 15 min. The maximum rotational speed was 4000 min^−1^, which resulted in a maximum compressive yield stress of about 50,000 Pa. The temperature was kept at 20 °C throughout all experiments.

For all experiments, a rectangular polycarbonate cuvette with a diameter of 10 mm was used. The sample volume was 1.5 mL, leading to a sample height $H_{0}$ of approximately 20 mm. The exact sample height for each sample was determined by the meniscus, which can be detected in the transmission profile (see Figure 1a). The sediment-liquid interface was detected as a threshold of 20% light transmission. The sediment bed height was then calculated as the difference between cuvette bottom ($R_{max}=129.5\;\mathrm{mm})$ and the sediment-liquid interface. For further interpretation and calculations, the sediment bed height $H_{\infty }$ at equilibrium state was considered.

**Figure 2:**
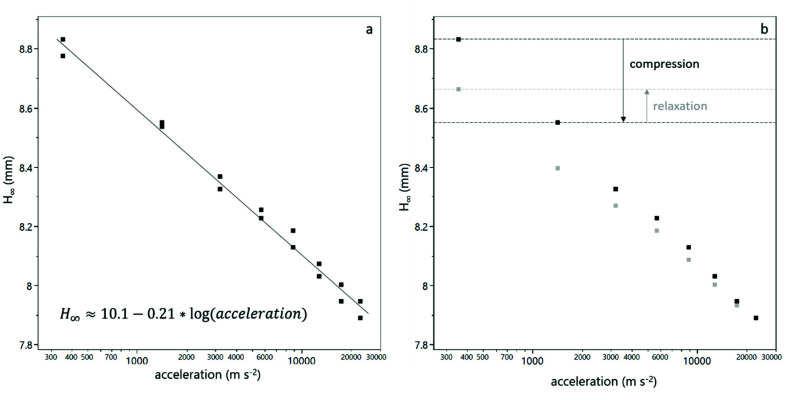
Equilibrium sediment bed height as a function of the centrifugal acceleration. The decreasing bed height with increasing acceleration can be fitted as a semi-logarithmic function for each duplicate measurement (**a**). After a step with reduced acceleration, the sediment bed height increases, which indicates a relaxation of the sediment (**b**). The black dots refer to values obtained from compression steps and grey dots to values obtained from decompression steps. The ratio of relaxation to compression is used to calculate the relaxation factor according to Equation (4).

All sediment bed heights $H_{\infty }$ showed a semi-logarithmic relationship with the centrifugal acceleration a, according to Equation (3). An exemplary fit is shown in Figure 2a.

(3) $$H_{\infty }=y-m\cdot \log\left(a\right)$$

The intercept y is a measure for the bed height at 1 m·s^−2^ centrifugal acceleration, i.e., nearly zero compression. The ratio of y to the initial sample height $H_{0}$ can then be considered as the relative bed height at zero compression. The slope m is a measure of the compressibility of the sediment.

The equilibrium bed height of each step i with a reduced acceleration (downward step) was used to calculate a relaxation factor rf. Therefore, the ratio of the bed height difference of the decompression step i and the bed height difference of the respective compression step i was calculated according to Equation (4). A schematic representation of this relaxation factor is also given in Figure 2b.

(4) $$f=\frac{H_{\infty ,\;decompression\;i}-H_{\infty ,\;compression\;i+1}}{H_{\infty ,\;compression\;i}-H_{\infty ,\;compression\;i+1}}$$

In order to obtain a measure for the deposit porosity, the equilibrium particle volume fraction in the sediment $\Phi_{base}$ can be calculated based on initial particle volume fraction $\Phi_{0}$, initial sample height $H_{0}$, radial position of the cuvette bottom $R_{max}$ and centrifugal acceleration a, according to Equation (5) [24,25].

(5) $$\Phi_{base}=\frac{\Phi_{0}\cdot H_{0}\cdot \left[1-\frac{1}{2\cdot R_{max}}\cdot \left(H_{\infty }+a\cdot \frac{dH_{\infty }}{da}\right)\right]}{\left(H_{\infty }+a\cdot \frac{dH_{\infty }}{da}\right)\cdot \left(1-\frac{H_{\infty }}{R_{max}}\right)+\frac{H_{\infty }^{2}}{2\cdot R_{max}}}$$

In order to determine the factor $\frac{dH_{\infty }}{da}$, the semi-logarithmic relationship between bed height and centrifugal acceleration was fitted, according to Equation (3). The slope m of this relationship was then used to determine $\frac{dH_{\infty }}{da}$ according to Equation (6).

(6) $$\frac{dH_{\infty }}{da}=\frac{-m}{a}$$

The respective yield stress, which contributes to sediment compression, can then be calculated according to Equation (7), where Δρ is the density difference between particle and surrounding liquid and $\Phi_{base}$ is the particle volume fraction in the sediment, as determined by Equation (5).

(7) $$P_{y}\left(\Phi_{base}\right)=\Delta \rho \cdot a\cdot \Phi_{base}\cdot H_{0}\cdot \left(1-\frac{H_{0}}{R_{max}}\right)$$

### 2.4. Dead-End Filtration

For dead-end filtration, an Amicon 8050 filtration cell (Merck KGaA, Darmstadt, Germany) with a sample volume of 50 mL and a membrane diameter of 44 mm was used. The filtration cell was equipped with a Whatman N1 filter paper (Whatman, Kent, UK) as a support layer and a 0.1 µm MF-Millipore™ membrane made from mixed cellulose esters (Merck KGaA, Darmstadt, Germany) as a selective layer. Before filtration, the membrane was flushed with pure water (dynamic viscosity $\eta_{w}$) at 0.5 bar transmembrane pressure $\Delta p_{TM}$, where the flux J was gravimetrically determined and subsequently used to calculate the membrane resistance $R_{m}$ according to Equation (8).

(8) $$J=\frac{\Delta p_{TM}}{\eta_{w}\cdot R_{m}}$$

50 mL yeast-BSA suspension was then placed in the pre-wetted filtration cell. The cell was pressurized with nitrogen, using a digital electronic pressure control unit (EL-PRESS, Bronkhorst, Ruurlo, The Netherlands). For each filtration, 10 g of permeate were collected, before the filtration was aborted. Due to the high dry matter content, 10 g of permeate was enough to build up a deposit layer of several gram weight and several mm thickness, sufficient for dry matter content determination. The flux was monitored by an electronic balance (Sartorius LA1200 S, Göttingen, Germany) with a measuring interval of 5 s. The BSA content in feed and permeate was analyzed by RP-HPLC according to Weinberger and Kulozik [7]. The transmission of BSA was calculated as the ratio of the BSA concentration in the permeate to the BSA concentration in the feed. The specific filtration resistance $\alpha_{av}$ was calculated according to Weinberger, et al. [26], where the values between 8 and 10 g of collected permeate were considered in order to represent the fully developed deposit layer.

### 2.5. Data Handling and Statistics

Each experiment was conducted as a randomized duplicate. In graphic illustrations, these duplicates are either given with both original values, a mean with the range indicated as error bar, or the result from fitting a model equation with the confidence interval of the fitting indicated as the error bar. In order to compare different milieu conditions, an analysis of variance was conducted and connecting letters are obtained from a student’s *t*-test with a 95% confidence interval. All calculations, statistical analysis, fittings and plotting were conducted with JMP^®^ Pro 14.1.

## 3. Results and Discussion

### 3.1. Compression of Yeast Deposits during Analytical Centrifugation

**Figure 3:**
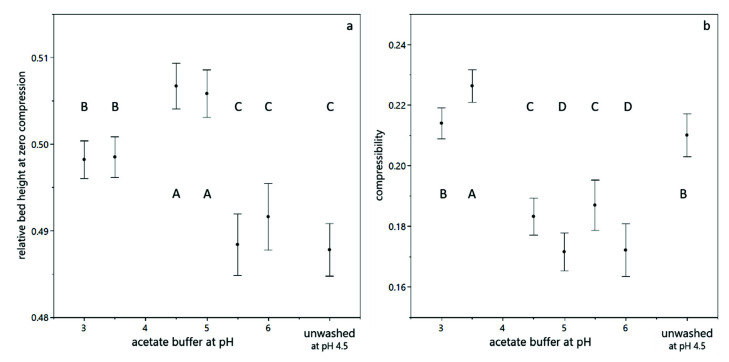
Compression behavior of yeast suspensions, calculated according to Equation (3). The relative bed height at zero compression (**a**) and the compressibility upon centrifugal acceleration (**b**) as a function of the buffer and its pH value. The error bars indicate the standard deviation from fitting the model equation. The connecting letters are obtained from a student’s *t*-test with a 95% confidence interval.

The focus of this work was to assess the compression and decompression behavior of yeast cell deposits by means of an analytical centrifuge and to determine the effect of milieu conditions on the deposit layer properties, by influencing cell-cell interactions as well as cell-protein interactions. As can be seen from Figure 1c, the compression and relaxation of the sediment in the analytical centrifuge was detectable and qualitatively recognizable. From Figure 2 it becomes clear that the bed height decreases with increasing centrifugal acceleration and corroborates the semi-logarithmic relationship of bed height and centrifugal acceleration. From this relationship, the relative bed height at zero compression and the compressibility of the sediment can be calculated (see Figure 3).

The relative bed height at zero compression was highest at pH 4.5 and pH 5.0, which indicates a loose deposit structure. At pH 3.0 and pH 3.5, the packing density without pressure application is higher due to the proximity to the isoelectric point of the yeast cells (between 3.0 and 4.0 [27]). The lowest relative bed height at zero compression was found for pH 5.5 and pH 6.0, which indicates an already dense packing of the sediment, even without any further compressive forces. This effect is not in line with the increasing negative charge of the cells, which should result in repulsion and thus a higher bed height. However, the ionic strength of the acetate buffer increases with increasing pH, which leads to a reduction of the Debye length ($\lambda_{D,\;pH\;3}=7.37\;\mathrm{nm}$, $\lambda_{D,\;pH\;4}=2.42\;\mathrm{nm}$, $\lambda_{D,\;pH\;5}=1.21\;\mathrm{nm}$, $\lambda_{D,\;pH\;6}=0.99\;\mathrm{nm}$) and can therefore increase packing density. The relative bed height at zero compression of unwashed yeast cells at pH 4.5 was lower than for washed yeast cells at the same pH, which can be attributed to the presence of salts, sugars and extrapolymeric substances, which act as mediators and linkers between single cells.

The compressibility under centrifugal acceleration is highest at pH 3.5. Considering the proximity of this pH value to the isoelectric point of yeast, this finding can be well explained by the reduced electrostatic repulsion. The second highest compressibility was found for pH 3.0 (still close to the isoelectric point) and for unwashed yeast cells, which can be explained by the presence of extracellular polymeric substances, which act as linking mediator between yeast cells and can fill voids in the packing structure. The lowest compressibility was found for washed yeast cells between pH 4.5 and pH 6, where the cells have a negative surface charge, which is opposed to the compressing forces. The increasing ionic strength with increasing pH value does not result in an increased compressibility, as after the initial packing at low compressive forces, the actual Debye length does not play a role in the subsequent compression.

**Figure 4:**
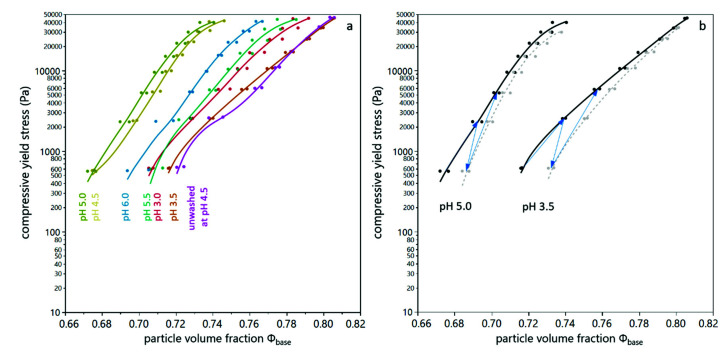
Compressive yield stress and particle volume fraction in the sediment during analytical centrifugation of yeast suspension for different pH values and buffers (**a**). The progression of compression and relaxation are indicated as a change of compressive yield stress and particle volume fraction in the sediment (**b**), where black dots indicate values after the compression step, grey dots indicate values after the decompression step and blue arrows indicate the chronological order of the single steps. The values of each duplicate measurement are given as individual dots. The lines are a guide to the eye.

In order to combine the bed height at zero compression and the compressibility with increasing acceleration, the particle volume fraction in the sediment (according to Equation (5)) can be determined for the different suspensions as a function of the compressive yield stress (according to Equation (7)). It can be seen in Figure 4 that the particle volume fraction in the sediment is highest for the unwashed yeast suspension, followed by suspensions with a low pH value, close to the isoelectric point. The lowest particle volume fraction in the sediment was found for pH 4.5 and pH 5.0, where the relative bed height at zero compression was high and the respective compressibility low. At pH 5.5 and pH 6.0, the compression was low as well, but the relative bed height at zero compression was also little, which results in a higher particle volume fraction in the sediment in comparison to suspensions at pH 4.5 and pH 5.0.

**Figure 5:**
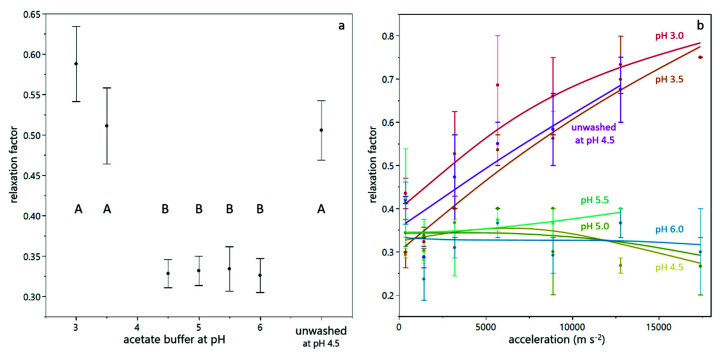
Relaxation behavior during analytical centrifugation of yeast suspensions. The mean relaxation factor depends on the buffer pH (**a**). The error bars indicate the standard error over all values from different centrifugal acceleration and a duplicate measurement. The connecting letters are obtained from a student’s t-test with a 95% confidence interval. For sediments in the connecting letter group (A), the relaxation factor depends on the centrifugal acceleration, as shown in (**b**).

In order to quantify the reversibility of compression, the relaxation factor was calculated according to Equation (4). Figure 5a shows the relaxation behavior for suspensions prepared with different buffers. It can be seen that the relaxation factor at low pH value and for unwashed yeast cells was higher than for suspensions at pH between 4.5 and 6.0. Figure 4b illustrates this difference in the compressive yield stress as a function of particle volume fraction for two different pH values. It is evident that at pH 3.5, the reversibility is higher than at pH 5.0, where the particle volume fraction decreases only slightly at reduced levels of centrifugal acceleration. From Figure 4b it also becomes clear that the particle volume fraction in the sediment of the suspension at pH 3.5 is above 0.74, which is considered as the densest packing density for spheres. In order to reach those high packing densities deformation of the cells is necessary. This deformation provokes a strong counterforce, which enables relaxation [28]. The fact that for sediments with high particle volume fractions the relaxation factor increases with increasing acceleration while it is independent for sediments with lower particle volume fractions (see Figure 5b) corroborates this hypothesis. The partial relaxation observed for yeast-only suspensions is in line with the reversibility of deposit layers from yeast cells observed during dead-end filtration by Ye and Chen [12].

### 3.2. Deposit Layer Formation and Filtration Performance

**Figure 6:**
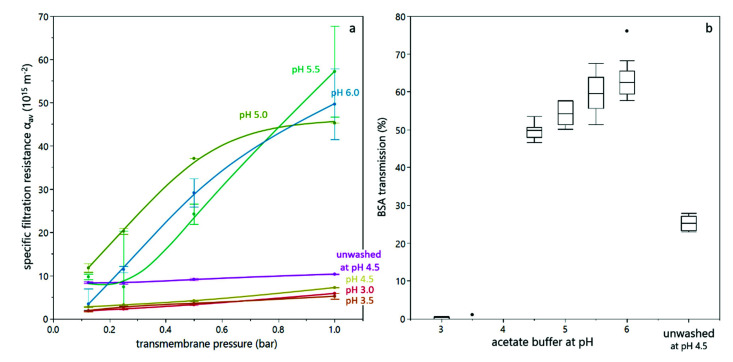
Dead-end filtration of a yeast-BSA mixture with 10% dry matter content: The specific filtration resistance as a function of transmembrane pressure, where error bars indicate the range of a duplicate (**a**) and the transmission of BSA for different buffers, where values from duplicate measurements at different transmembrane pressure were considered (**b**). The BSA transmission did not significantly change with transmembrane pressure in the investigated transmembrane pressure range.

In order to better assess the correlation between sediment formation in the analytical centrifuge and deposit layer formation during filtration, dead-end filtration trials were conducted. The yeast suspension was supplemented with BSA as a model protein. By means of BSA, the effect of deposit formation on the transmission of solutes was assessed as filtration efficiency criterion besides the filtration resistance and deeper insights regarding the structure and retentive effect of deposited cells was derived. Dead-end filtration trials were conducted at different transmembrane pressures (in a similar range as the compressive forces in the analytical centrifuge) and different buffer pH values for washed and unwashed yeast cells. All experiments were conducted with 5 and 10% dry matter content, but the results were alike. The specific filtration resistance for duplicate trials is shown in Figure 6a. Firstly, it can be seen that the resistance at lower pH was significantly lower than at high pH, and increased less with increasing transmembrane pressure. This is in accordance with literature [12,20], but contradictory to the findings from analytical centrifugation, which showed a high compressibility at low pH value. This discrepancy might be attributed to the presence of BSA, as discussed below (see Figure 8). The very pronounced difference between pH 4.5 and pH 5.0 can also be attributed to BSA. The isoelectric point of BSA is at pH 4.7, i.e., between the two experimental settings. Thus, at pH 4.5, BSA is slightly positively charged and interacts with negatively charged yeast cells, leading to a different deposit layer structure than at pH 5.0, where both BSA and yeast cells are negatively charged. Secondly, the resistance for unwashed yeast cells was significantly higher than for washed yeast cells at the same pH value, which is in accordance with literature [21] and also with findings from analytical centrifugation (see Figure 3 and Figure 4).

Figure 6b shows the transmission of BSA as a function of the buffer pH. It can be seen that at pH 3.0 and pH 3.5, almost no BSA was found in the permeate. This is due to the reduced electrostatic repulsion between BSA and yeast cells, between BSA and the membrane, as well as between BSA molecules, due to the proximity of the isoelectric points of BSA and yeast cells. Ye and Chen [12] and Foley [23] already postulated the formation of an open-porous structure due to aggregation of particles or molecules at their isoelectric point, which is in accordance with our findings. For pH values above 4.5, the BSA transmission was above 50% and increased with increasing pH value. As the specific filtration resistance and compression behavior is similar for these pH values, this effect is probably rather attributed to adsorption, which decreases with increasing pH value, where electrostatic repulsion is more pronounced. The compressive effect of increasing transmembrane pressure cannot be observed in the transmission data. Also, the transmission of BSA of an unwashed yeast suspension is significantly lower than for a washed yeast suspension, which is in accordance with the findings for higher compressibility and higher filtration resistance in the presence of extracellular polymeric substances, which can act as mediator between yeast cells.

**Figure 7:**
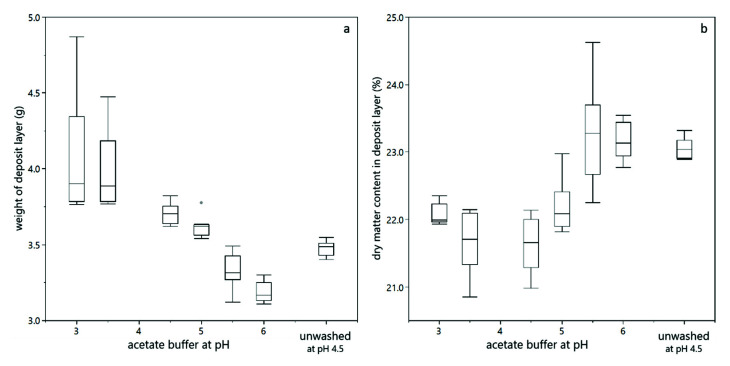
Properties of the deposit layer formed during dead-end filtration of a yeast-BSA mixture with 5% dry matter content: The final weight of the deposit layer including the membrane (**a**) and the dry matter content of the deposit (**b**). All duplicate filtration trials at different transmembrane pressure were considered. The transmembrane pressure had no significant effect on the weight and dry matter content of the deposit layer. Results for the 10% dry matter suspension are comparable, but with a reduced precision of the deposit layer weight, due to handling issues with the quite high deposit.

In order to gain more insights into the deposit layer character, the deposit layer formed during filtration was weighed and the dry matter content in the deposit layer was analyzed. In our previous work we have shown that heavier deposit layers contained more water. Thus, the deposits were more porous and therefore better permeable than dense deposit layers containing less water with a lower weight [26]. Figure 7 shows that deposit layers formed at pH 3.0 and pH 3.5 were hydrated, leading to a high deposit layer weight, but a low dry matter content. This fits well with the low filtration resistance observed. With increasing pH, the deposit layer weight decreased and the dry matter content of the deposit layer increased. Again, this fits qualitatively well with filtration resistances obtained. Finally, also the effects observed for unwashed yeast cells could be confirmed, i.e., a lower weight and a higher dry matter content of the deposit layer in comparison to washed yeast cells at the same pH value. Thus, unwashed yeast cells lead to a more dense and less permeable deposit layer due to the presence of extracellular polymeric substances.

**Figure 8:**
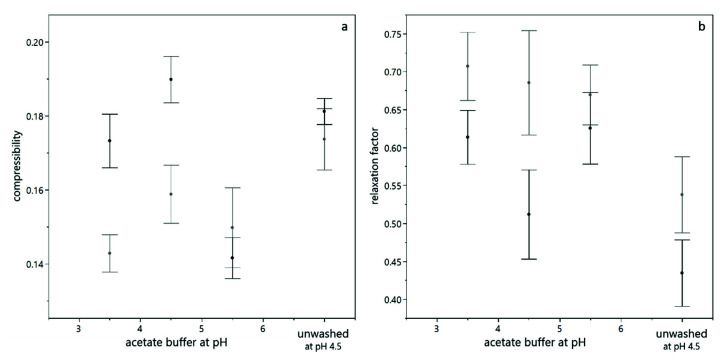
Compression behavior of yeast suspensions with (grey) and without (black) BSA present. The compressibility is given for different buffer pH, where the error bars indicate the standard deviation from fitting the model equation (**a**). The relaxation behavior of the sediments is given for different buffer pH, where the error bars indicate the standard error over all values from different centrifugal acceleration and a duplicate measurement (**b**).

As described above, the results obtained from analytical centrifugation are only partly in line with the results obtained by dead-end filtration. However, analytical centrifugation was performed with yeast only suspensions and filtration trials were conducted with yeast and BSA mixtures. As shown in Figure 6, interaction between BSA and yeast cells play a significant role, especially at low pH values. In order to assess whether these interactions provoked the differences observed, analytical centrifugation was performed for yeast suspensions with and without BSA added. Figure 8 shows the compressibility and relaxation factor obtained from these trials. It can be seen that the presence of BSA reduces the compressibility and increases the relaxation at low pH (pH 3.5 and pH 4.5). At pH 5.5, where no interaction between BSA and yeast cells is expected due to electrostatic repulsion, the presence of BSA did not alter the compression and relaxation behavior of the sediments. For unwashed suspensions, the effect was less pronounced than for washed yeast cells, probably due to the more complex milieu including extracellular polymeric substances. Thus, analytical centrifugation and filtration results are in accordance when model systems of the same composition are compared.

### 3.3. Comments on the Applicability of Analytical Centrifugation

In comparison to flux or pressure stepping experiments, the analytical centrifugation method only requires sample volumes of a few milliliter and the execution takes only few minutes of manual work. With the analytical centrifuge used in this work, eight samples can be analyzed simultaneously. However, in order to reach accurate and precise results, some effort has to be put in the choice and reproducibility of the dry matter content in comparison to the direct evaluation of filtration performance. Firstly, the dry matter content has to be chosen high in order to reach an adequate bed height and thus an adequate resolution. The radial resolution of the LUMiFuge is limited to 0.014 mm. This at first appears sufficient, but when the bed height difference between different compression steps is only between 0.1 and 0.2 mm (and the relaxation effect even below this level), the radial resolution introduces an experimental uncertainty, which should be minimized by choosing high dry matter contents. Secondly, the precise dry matter content has a big impact on the compressive yield stress and the particle volume fraction in the sediment and therefore has to be set as accurate as possible. If those two points are taken into account, the presented method can be successfully applied as a screening method for process optimization, even when only small differences in compressibility and relaxation behavior are expected. In addition, the deposit layer forming material subject to investigation should be sedimentable particulate matter and be present in the complex environment used for filtration. It should be noted that a forecast on solute transmission can only be deduced from the compressibility data as long as no significant interactions between solute and particle occur.

## 4. Conclusions

In this work, we have shown the applicability of analytical centrifugation as a screening method for compression and relaxation analysis of deposits formed during microfiltration processes. The impact of buffer pH value and the presence of extracellular polymeric substances, which both influence the filtration resistance, could be simulated by analytical centrifugation. The presence of BSA as a model solute also had a tremendous effect on compression and relaxation behavior. Considering the fact that results from analytical centrifugation were closer to results from filtration experiments when the exact same composition of the feed was used, we are convinced a transfer of this method to real fermentation broths, i.e., complexly composed feed systems, is likely to be successful. Using analytical centrifugation as a screening method can enhance process optimization for filtration processes (mostly regarding flux, but if no significant interactions between target solutes and particles occurs, transmission can be deduced from analytical centrifugation as well). For instance, process conditions can be chosen accordingly to the compression behavior of the retained cells, i.e., high transmembrane pressure for incompressible deposits and low transmembrane pressure for highly compressible deposits. Furthermore, milieu conditions of a given feed suspension can be adjusted in order to increase deposit reversibility and thus maximize the efficiency of fouling mitigation techniques, such as backflushing or alternating crossflow.

By consulting compressibility differences between suspensions containing BSA and lacking BSA and taking into account the close to zero transmission of BSA at low pH values, this work also helps to better understand the role of yeast cell-BSA interaction on the stability and porosity of deposit layers.

## Data Availability

CThe datasets generated during and/or analyzed during the current study are available from the corresponding author on reasonable request.
